# The total wrist arthroplasty

**DOI:** 10.1186/1753-6561-9-S3-A86

**Published:** 2015-05-19

**Authors:** Amit Gupta

**Affiliations:** 1 Department of Orthopedic Surgery, University of Louisville, Kentucky, 40292, USA

## 

Traditional management of wrist arthritis consists of proximal row carpectomy, partial carpal fusions, or, in the event of pancarpal arthritis, total wrist fusion. Although proximal row carpectomy and partial wrist fusions preserve some motion at the wrist while relieving pain symptoms, the quality of results obtained from these procedures is not predictable or optimal in many instances. Total wrist fusions are credited with universal pain relief at the expense of wrist motion. However, critical analysis of the results of total wrist arthrodesis shows that the results are not always consistent with what has been claimed and that pain relief is unpredictable at best. In a recent study, 14 of 22 patients who had undergone wrist arthrodesis had residual pain, and 4 of these patients had severe pain 6 years later. In another study, only 6 of 36 patients remained pain-free 4 years after wrist arthrodesis.

Moreover, many patients do not like loss of motion in their wrists. In a study of wrist arthrodesis, only 40% of patients were satisfied at 1 year. In another study, 100% of patients who had wrist arthrodesis indicated that they would have a procedure performed so they could move their wrists again.

Management of hip, knee, ankle, and shoulder joints has evolved from arthrodesis to arthroplasty. The wrist joint awaits the same pattern of evolution with the advent of reliable designs.

In this presentation, I will first describe the design rationale of the Stryker ReMotion Total Wrist arthroplasty along with historical antecedents. Next, I will outline the technique of implantation with tips and tricks for special situations. Finally, I will discuss 10-year results and comment on future prospects of total wrist arthroplasty.

